# Measuring Distortion-Product Otoacoustic Emission With a Single Loudspeaker in the Ear: Stimulus Design and Signal Processing Techniques

**DOI:** 10.3389/fdgth.2021.724539

**Published:** 2021-09-01

**Authors:** Wei-Chen Hsiao, Yung-Ching Chen, Yi-Wen Liu

**Affiliations:** Department of Electrical Engineering, National Tsing Hua University, Hsinchu, Taiwan

**Keywords:** hearing, otoacoustic emissions, intermodulation distortion, nonlinear signal processing, Volterra filtering

## Abstract

The distortion-product otoacoustic emission (DPOAE) is a backward propagating wave generated inside the cochlea during the wave amplification process. The DPOAE signal can be detected rapidly under relatively noisy conditions. In recent years, the earphone industry demonstrated interest in adopting DPOAE as an add-on feature to make their product “intelligent” of inner-ear status. However, a technical challenge remains to be tackled—the loudspeaker in an earphone generates its own cubic distortion at the same frequency as DPOAE. Unfortunately, the intensity of loudspeaker distortion is typically comparable to that of the DPOAE, if not higher. In this research, we propose two strategies, namely *compensation* and *cancellation*, to enable DPOAE measurement with a single loudspeaker. The compensation strategy exploits the part of the growth function of the loudspeaker distortion which is almost linear, and thus suppresses the distortion it generates while retaining a larger portion of DPOAE in the residual signal. The cancellation strategy utilizes a one-dimensional Volterra filter to remove the cubic distortion from the loudspeaker. Testing on normal-hearing ears shows that the compensation strategy improved the DPOAE-to-interference ratio by approximately 7 dB, resulting in a cross-correlation of 0.62 between the residual DPOAE level and the true DPOAE level. Meanwhile, the cancellation strategy directly recovered both the magnitude and the phase of DPOAE, reducing the magnitude estimation error from 15.5 dB to 3.9 dB in the mean-square sense. These pilot results suggest that the cancellation strategy may be suitable for further testing with more subjects.

## 1. Introduction

Otoacoustic emissions (OAEs) are sounds generated in the cochlea that propagate backward to emit from the ear (1). OAEs can be classified into two types (2)—spontaneous OAEs (SOAEs) and evoked OAEs. SOAEs occur in the absense of external stimulus, and evoked OAEs can be regarded as acoustic responses to external stimulus. Within the family of evoked OAEs, the distortion-product OAE (DPOAE) is widely used as an objective tool for detecting hearing impairment associated with outer hair cell (OHC) dysfunctions (3, 4). To measure DPOAE, a pair of primary tones at frequencies *f*_1_ < *f*_2_ are delivered to an earphone inserted to the ear canal. With appropriately chosen intensities and frequencies of the primary tones [e.g., *f*_2_/*f*_1_ = 1.22, (5)], the most prominent distortion product would occur at 2*f*_1_ − *f*_2_ and it can be recorded from a microphone in the ear canal. Because the primary tones' excitation patterns mainly overlap near the *f*_2_ characteristic place in the cochlea (6), the sound-pressure level (SPL) of DPOAE at *f*_DP_ = 2*f*_1_ − *f*_2_ represents the cochlea's ability to process signals normally at frequency *f*_2_. Thus, DPOAE serves as a robust and non-invasive tool for assessing cochlear functions in a frequency-specific manner (4). It has been applied clinically for hearing screening (7, 8), and diagnosis of acute hearing loss (9) and other kinds of hearing impairment (10, 11).

Typically, a clinical DPOAE probe consists of two loudspeakers and one microphone; for each ear, the primary tones at *f*_1_ and *f*_2_ are separately delivered to the two speakers to avoid generating intermodulation distortion (IMD) electrically (12). As a rare exception, a single-speaker configuration was adopted for measuring vibration caused by DPOAE on insect tympanal organs (13); however, it was emphasized that one should avoid over-driving the speaker and thus producing IMD artifacts (14). In the field of cochlear neurophysiology, nonetheless, a combination of 5–7 tones with carefully arranged frequencies could be delivered simultaneously to a single speaker to elicit auditory-nerve responses (15); in their study, loudspeaker IMD was not a concern because the neural response by nature contains strong quadratic-distortion components which actually facilitate efficient estimation of the cochlear tuning curve at the auditory-nerve level.

Recently, the two-loudspeaker hardware design has been adopted by a commercial headphone that promotes at-home DPOAE measurement as a means of providing personalized frequency response adjustment (16). The two-speaker design seems necessary because, even with a high-quality headphone or earphone, the total harmonic distortion (THD) can reach 3% when driven to its full dynamic range (17). This THD level is acceptable for listening to music; however, when delivering two pure tones simultaneously, we found that the distortion generated by such speakers would significantly interfere with the DPOAE from the ear since the cubic distortion of the speaker also occurs at *f*_DP_.

Nevertheless, human DPOAE and loudspeaker IMD have different generation mechanisms even though they may occur at the same frequency. For example, the DPOAE signal is comprised of a direct component plus a reflective component (18, 19); the direct component travels back from the *f*_2_ characteristic place in the cochlea, while the reflective component travels further to the *f*_DP_ place and changes direction due to *coherent reflection* (20). The two components thus have different latency in the range of 5–20 ms, which allows them to be separated via envelope-tracking techniques (21). Also, they may superpose constructively or destructively depending on their relative phase. In comparison, the loudspeaker IMD is perhaps elicited nearly instantaneously, so we expect that its latency and rate of growth with respect to the primary tone levels *L*_1_ and *L*_2_ might differ from that of DPOAE. In this research, we seek to exploit these differences and develop methods for estimation of DPOAE levels using a *single speaker*, despite of interference from loudspeaker IMD.

In particular, we propose stimulus design and signal processing strategies that handle the interference issues due to loudspeaker IMD. The first strategy is called *compensation* and it involves finding a combination of *L*_1_ and *L*_2_ such that the IMD level grows *almost linearly* with respect to simultaneous increment in (*L*_1_, *L*_2_). The second strategy is called *cancellation* and it utilizes 3rd-order one-dimensional Volterra filter (22) to subtract the loudspeaker IMD from the signal. The organization of the remaining part of this paper follows the standard order of Methods, Results, Discussion, and Conclusion.

## 2. Methods

In this section, we first review the mathematics of IMD generated by two tones. Typical spectrums of DPOAE and loudspeaker IMD will be shown so we can examine the similarities and differences. Then, the compensation strategy and the cancellation strategy will be described. This section ends with brief descriptions of the recording equipment and the human subjects who participated in the testing.

### 2.1. IMD and Mathematical Notations

Assuming that an acoustic or electrical stimulus, called the input signal, contains two frequency components *f*_1_ and *f*_2_, so the signal can be expressed as follows,


(1) $$\begin{array}{l} x\left(t\right)=A_{1}\cos\left(2\pi f_{1}t+\phi_{1}\right)+A_{2}\cos\left(2\pi f_{2}t+\phi_{2}\right), \end{array}$$

where *A*_1_, *A*_2_ and ϕ_1_, ϕ_2_ denote the amplitude and phase for two components, respectively. Assume that the stimulus is delivered to a nonlinear system *G* so that the response *y*(*t*) can be denoted as *y*(*t*) = *G*(*x*(*t*)). When *G* is instantaneous, it can be expanded by Taylor's series near the origin; that is,


(2) $$\begin{array}{l} G\left(\eta \right)=G\left(0\right)+\overset{\infty }{\underset{k=1}{\sum }}\frac{G^{\left(k\right)}\left(0\right)}{k!}\eta^{k}. \end{array}$$

**Figure 1:**
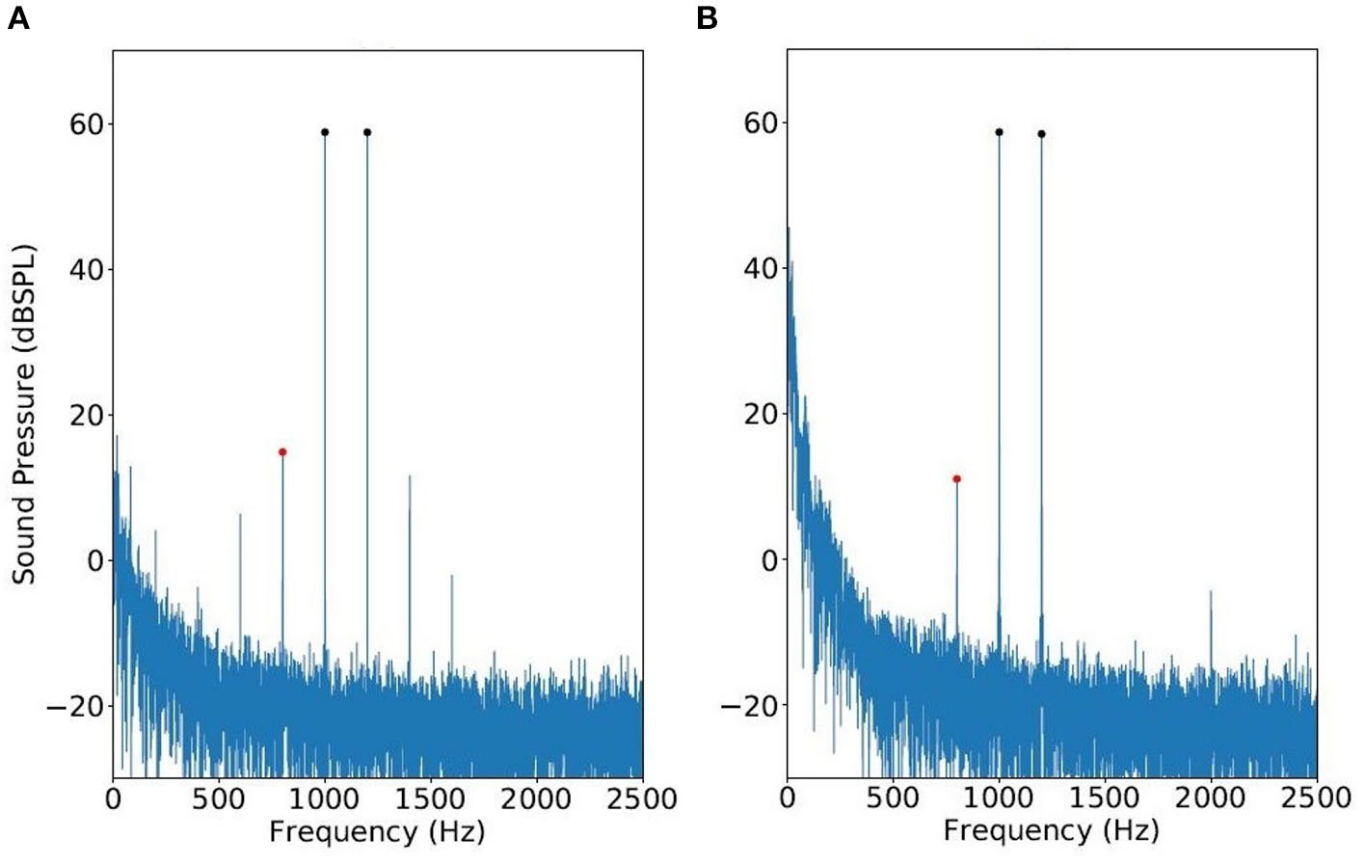
Spectrums of modulation distortion produced by a single speaker **(A)** vs. true DPOAE obtained with a two-speaker probe **(B)**. The primary frequencies are *f*_1_ = 1, 000 Hz and *f*_2_ = 1, 200 Hz.

By setting η = *x*(*t*) and through simple trigonometry, one can show that


(3) $$\begin{array}{l} y\left(t\right)=\text{Re}[\underset{m}{\sum }\underset{n}{\sum }B_{m,n}e^{j2\pi \left(mf_{1}+nf_{2}\right)t}], \end{array}$$

where *B*_*m,n*_ are complex-valued coefficients, and *m* and *n* sum over all integers such that *mf*_1_ + *nf*_2_ > 0. The components *B*_*m*, 0_ or *B*_0,*n*_ are referred to as harmonics; the additional components *B*_*m,n*_ when *m* and *n* are both nonzero are called the intermodulation products and they can be classified by their order |*m*| + |*n*|. In the context of DPOAE measurement with a single loudspeaker, *y*(*t*) thus contains not only the primary frequencies *f*_1_ and *f*_2_ but also higher-order components at *mf*_1_ + *nf*_2_. Empirically, the 3rd-order intermodulation products *B*_2,−1_ and *B*_−1,2_ are most prominent from a loudspeaker (see Figure 1A), and a few other components such as *B*_3,−2_, *B*_−2,3_ can also be identified above the noise floor. In comparison, from the DPOAE spectrum (measured by the conventional two-speaker approach), the *B*_−1, 2_ component corresponding to frequency 2*f*_2_ − *f*_1_ usually cannot be detected (see Figure 1B). The reason is because, even though the intermodulation product at 2*f*_2_ − *f*_1_ is generated due to OHC nonlinearity near the *f*_2_ place, it is prohibited from backward propagation along the basilar membrane (23).

By inspecting Figure 1, note that the loudspeaker components occurring at 2*f*_1_ − *f*_2_ has the same frequency as DPOAE. This 3rd-order component from the loudspeaker is referred to as IMD3 hereafter, and we shall investigate how to estimate DPOAE regardless of the presence of IMD3.

### 2.2. The Compensation Strategy

The DPOAE level, denoted as *L*_DP_, depends systematically on parameters (*L*_1_, *L*_2_, *f*_1_, *f*_2_). The relation *L*_DP_ = *L*_DP_(*L*_1_, *L*_2_, *f*_1_, *f*_2_) was comprehensively measured from a cohort of 20 normal-hearing human subjects (24) with a purpose to recommend the optimal choice of $L_{1}=L_{1}^{\text{opt}}$ that maximizes *L*_DP_ given *L*_2_. When *L*_1_ increases beyond $L_{1}^{\text{opt}}$, *L*_DP_ starts to decrease due to two-tone suppression (25). The same phenomenon has also been reproduced *in silico* by simulation of cochlear mechanics (23). In this section, we report on how differently the IMD3 level depends on the parameters, and hence devise a way to suppress IMD3 by considering two sets of primary-tone level (*L*_1_, *L*_2_) jointly.

#### 2.2.1. Growth Function of IMD3

**Figure 2:**
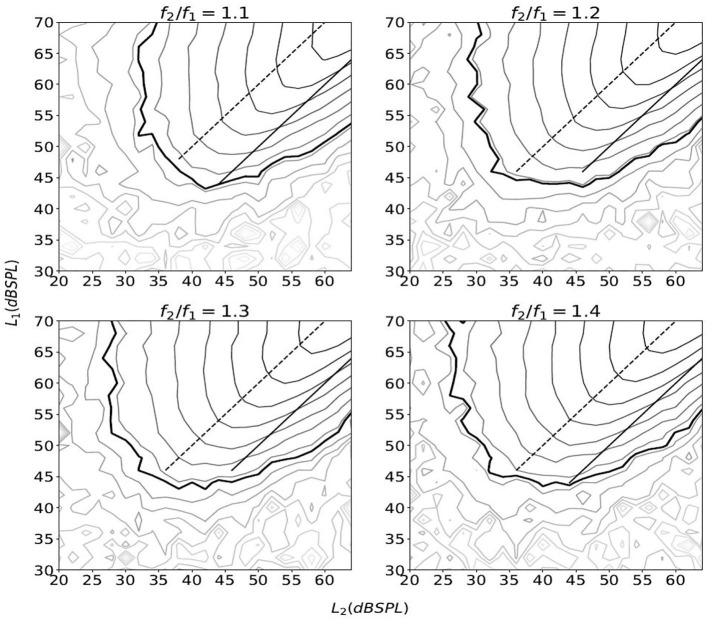
The equal-level contour plots of IMD3 as a function of (*L*_1_, *L*_2_). The thick line marks *L*_IMD3_=0 dB SPL, and other lines are in 4-dB steps. The IMD3 level were obtained by varying *L*_1_ from 30 to 70 dB SPL and *L*_2_ from 20 to 64 dB SPL in 2 dB steps. For each combination of (*L*_1_, *L*_2_), the stimulus lasted for 1.0 s with a 2.5-ms raised cosine ramp for the rising and falling edges. The stimulation was repeated five times and the average IMD3 level is shown. The solid line and the dashed line represent *L*_1_ = *L*_2_ and *L*_1_ = *L*_2_ + 10 (dB), respectively.

In contrast to cochlear mechanics, the loudspeaker nonlinearity does not demonstrate two-tone suppression; for instance, Figure 2 shows IMD3 level as a function of (*L*_1_, *L*_2_) with *f*_1_ = 1, 000 Hz and several different ratios *f*_2_/*f*_1_. The results were obtained by delivering the primary tones to one of the two loudspeakers of a DPOAE probe (see section 2.4 for details) and measuring the response inside a syringe of approximately 2.0 cc. For any fixed *L*_2_, as *L*_1_ increases, we do not find a clear $L_{1}^{\text{opt}}$ beyond which *L*_IMD3_ starts to decrease, and this is quite unlike what was observed in human subjects with the most commonly used frequency ratio *f*_2_/*f*_1_ ≈ 1.2 (24, Figure 1).

**Figure 3:**
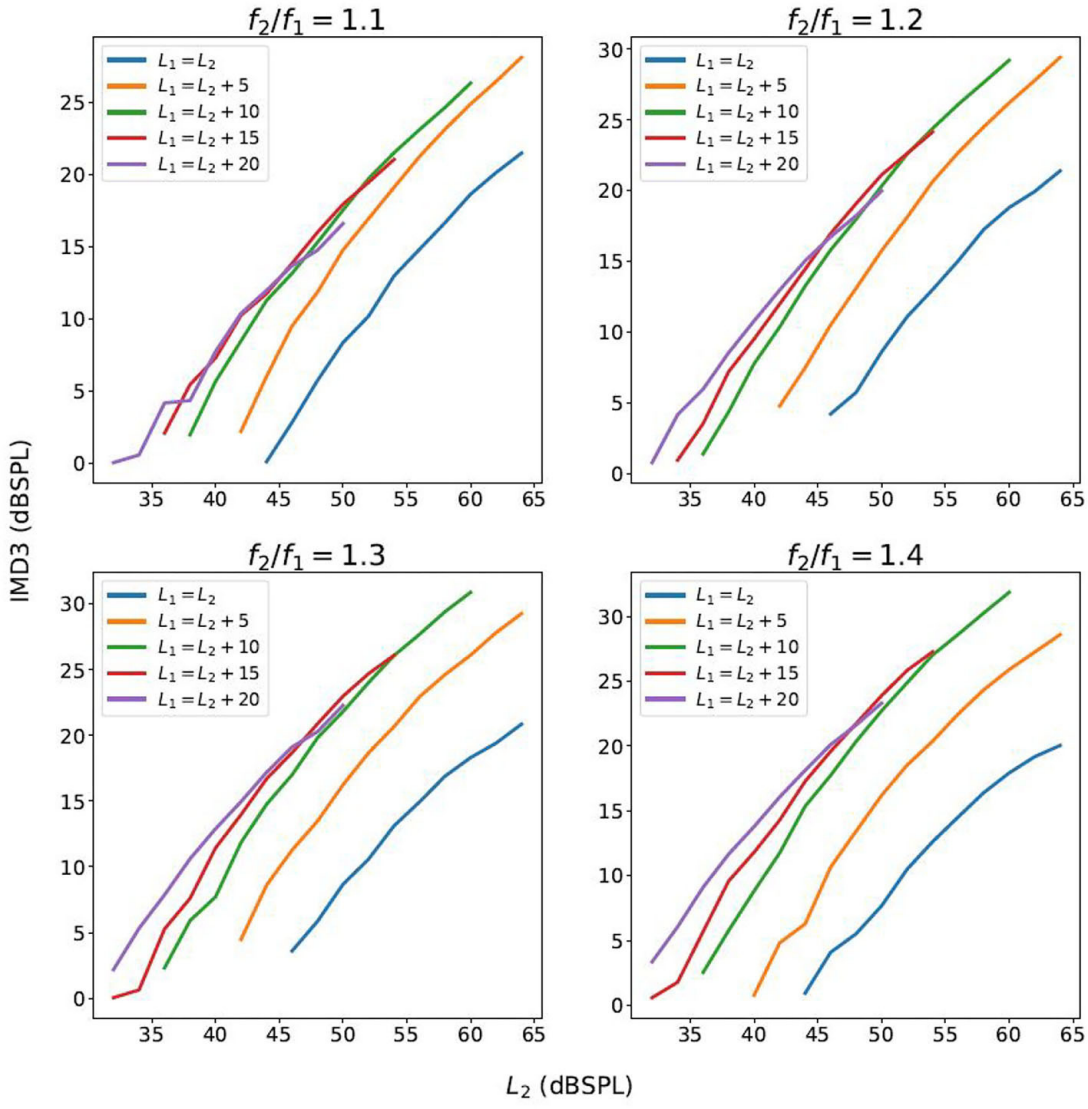
The growth function of the IMD3 level with respect to *L*_2_, as *L*_1_ and *L*_2_ increase proportionately.

**Figure 4:**
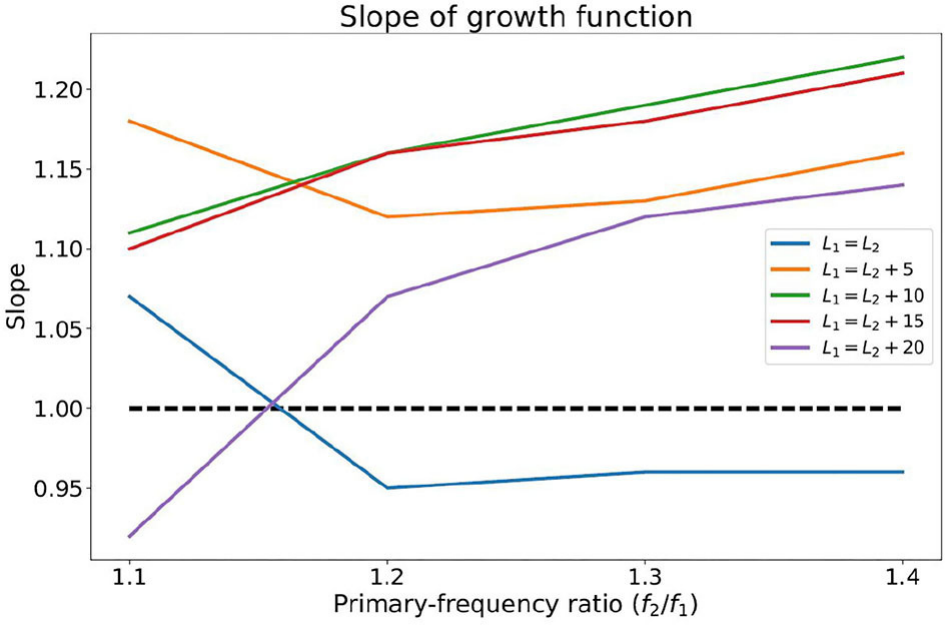
The slope of the growth functions of the IMD3 shown in Figure 3 with various primary-frequency ratios and primary-level differences.

The contour plot of IMD3 also differs from that of human DPOAE in the rate of growth with respect to *L*_1_ and *L*_2_. In particular, when *L*_1_ and *L*_2_ increases proportionately as they move toward the top-right corner of the plot along the straight lines *L*_1_ = *L*_2_ + 10 or *L*_1_ = *L*_2_, the rate of growth of *L*_IMD3_ with respect to *L*_2_ seems to be close to 1.0 dB/dB for a wide range of *L*_2_ and across different *f*_2_/*f*_1_ ratio (see the “growth functions” in Figure 3). The slope of these growth functions are shown in Figure 4, and the path *L*_1_ = *L*_2_ happens to have the slope that is closest to 1.0 dB/dB across different primary-frequency ratios. In comparison, the average human DPOAE growth rate when *L*_1_ = *L*_2_ falls in the range of 0.3 − 0.5 if *f*_2_/*f*_1_ ≈ 1.2 (24, Figure 1). By exploiting this difference between the growth function of loudspeaker IMD3 and human DPOAE, we present a method that suppresses the IMD3 level while partially retaining the DPOAE.

#### 2.2.2. Signal Acquisition Protocol

To leverage the part of IMD3 growth function that is almost linear (i.e., 1 dB/dB slope), we can devise the following performance metric,


(4) $$\begin{array}{l} J=\frac{\left|\beta G_{\text{DP}}\left(P_{1},P_{2}\right)-G_{\text{DP}}\left(\beta P_{1},\beta P_{2}\right)\right|}{\left|\beta G_{\text{IMD}3}\left(P_{1},P_{2}\right)-G_{\text{IMD}3}\left(\beta P_{1},\beta P_{2}\right)\right|}, \end{array}$$

where (*P*_1_, *P*_2_) denotes primary-tone sound pressure in Pa, β > 1 denotes a scaling factor, and *G*_IMD3_ and *G*_DP_ denote the sound pressure of IMD3 and DPOAE in Pa, respectively. Conceptually, the goal of the compensation strategy is to choose (*P*_1_, *P*_2_) and β such that *J* is maximized. However, since the numerator (referred to as *DPOAE residual*) would vary among individuals, we seek to minimize the denominator in *J*, referred to as the *IMD3 residual*. Based on the results shown in Figure 4, we selected *L*_1_ = *L*_2_ (i.e., *P*_1_ = *P*_2_) for the remaining parts of this paper. As implied by Figure 3, choosing *L*_1_ anywhere between 45 dB to 65 dB SPL should work well in reducing the IMD3 residual since *G*_IMD3_(β*P*_1_, β*P*_2_) ≈ β*G*_IMD3_(*P*_1_, *P*_2_). In contrast, we expect a larger proportion of DPOAE would remain in the DPOAE residual because the rate of growth against (*P*_1_, *P*_2_) is sub-linear (i.e., <1 dB/dB).

Based on the above-mentioned concept, we propose the following signal acquisition protocol.

- Step 1: Calibrate the stimulus levels *A*_1_ and *A*_2_ in Equation (1) such that *P*_1_ = *P*_2_ in the ear canal.
- Step 2: Transform the recorded signal to the frequency domain, and calculate the magnitude at 2*f*_1_ − *f*_2_, which is the vector sum of IMD3 and DPOAE. Denote the result as *Y*(*P*_1_, *P*_2_).
- Step 3: Repeat Step 2 with increased primary-tone levels β*P*_1_ and β*P*_2_. Denote the result as *Y*(β*P*_1_, β*P*_2_).
- Step 4: Calculate the magnitude of residual at 2*f*_1_ − *f*_2_, defined as |β*Y*(*P*_1_, *P*_2_) − *Y*(β*P*_1_, β*P*_2_)|.

To summarize, the goal of the signal acquisition protocol is to keep a large portion of DPOAE in the residual while maximally suppressing IMD3 at the same time.

### 2.3. The Cancellation Strategy

To describe the cancellation strategy, since digital adaptive filtering techniques are involved, we change the time variable from *t* to the integer index *n* (not to be confused with the index *n* in Equation 3). We follow a standard digital signal processing notation in defining *y*[*n*] = *y*(*nT*) (26), where *y*(*t*) is a continuous-time signal, *T* denotes the sampling period, and *y*[*n*] denotes the result after sampling in time.

**Figure 5:**
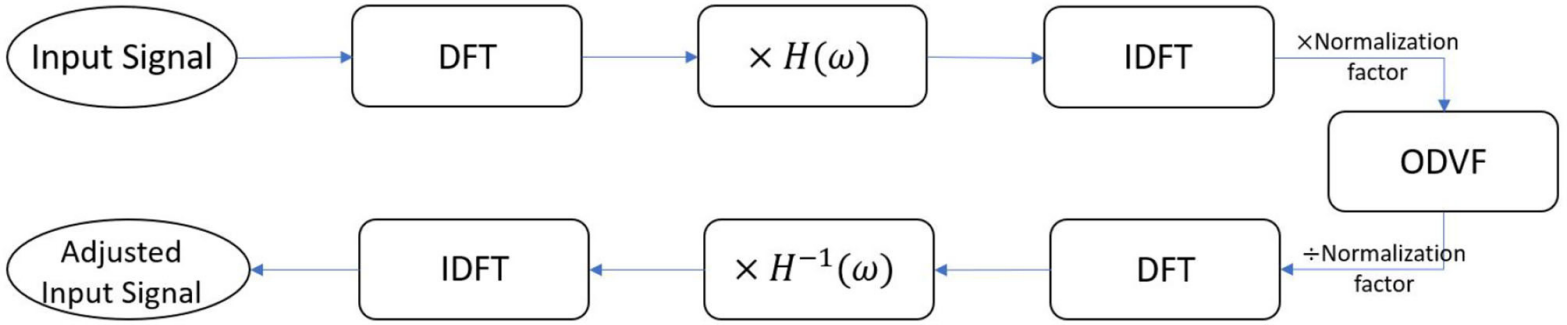
Block diagram of the IMD3 cancellation method. DFT, discrete Fourier transform; IDFT, inverse DFT; ODVF, one-dimensional Volterra filter. *H*(ω) represents the linear coupling from the speaker to the microphone, which was measured offline by a sweep-sine chirp method described in section 2.3.1.

The idea behind this strategy is to cancel IMD3 instantaneously. To achieve this goal, we utilize two techniques, namely a phase-controlled exponential swept-sine chirp and the one-dimensional Volterra filters (ODVFs), to adjust the input signal before sending it to the speaker. The workflow is shown in Figure 5 where *H*(ω) denotes a frequency response measured by the phase-controlled exponential swept-sine chirp (to be described in section 2.3.1), and the normalization factor ensures that the input to ODVF is limited to the range [−1, 1]. The input signal which contains two pure tones for DPOAE measurement is first transformed from time domain to frequency domain through DFT, then transformed back to time domain after multiplying with the frequency response of the single speaker *H*(ω). The filter coefficients of ODVF are meant to be obtained *offline* by adaptive LMS algorithm (to be described in section 2.3.2); when applied online, the ODVF filter coefficients are fixed. Subsequently, the multiplication by *H*^−1^(ω) is to compensate the gain and phase change due to multiplication by *H*(ω). This workflow produces an adjusted input signal to be delivered to the single speaker for the purpose of measuring DPOAE.

The rationale of this design is to artificially generate intermodulation distortions by the ODVF filter but with an inverted polarity, so they can cancel the real intermodulation distortion generated by the loudspeaker. We shall see in section 2.3.2 that the ODVF is trained off-line to emulate the situation of using two separate loudspeakers.

#### 2.3.1. Linear System Estimation by Phase Controlled Exponential Swept-Sine Chirp

A phase-controlled exponential swept-sine chirp (27) is used to obtain the linear coupling response from the loudspeaker to the microphone. This chirp exhibits an instantaneous frequency that increases exponentially with time as follows,


(5) $$\begin{array}{l} s\left[n\right]=A\sin\left(\frac{\pi L}{2^{Q}\ln\left(2^{Q}\right)}{\left(2^{\frac{Q}{N}}\right)}^{n}\right), \end{array}$$

where *A* is the amplitude of the sine wave, *Q* is an integer number of octaves, *L* is the ideal chirp length, and *N* is the real chirp length which is *L* rounded to the nearest integer. We also need an inverse chirp to convolve with, which can be expressed as


(6) $$\begin{array}{l} s^{-1}\left[n\right]=\frac{Q\ln\;2}{A^{2}\left(1-2^{-Q}\right)}\cdot s\left[N-n\right]{\left(2^{\frac{Q}{N}}\right)}^{-n}. \end{array}$$

The result of convolving *s*[*n*] and *s*^−1^[*n*] is approximately a Dirac delta impulse, and the gain is 0 dB for all frequencies.

We set the chirp's frequency to glide from 47 to 24, 000 Hz and the chirp length was 10.5 s. Then, we delivered the chirp to drive the single speaker and recorded the sound simultaneously. Subsequently, the recorded signal was convolved with the inverse chirp (Equation 6) to obtain the impulse response *h*[*n*] that characterizes the linear coupling from the speaker to the microphone. The Fourier transform of *h*[*n*] is denoted as *H*(ω) in Figure 5.

#### 2.3.2. IMD3 Cancellation by One-Dimensional Volterra Filtering

Since the intermodulation is due to loudspeaker nonlinearity, the behavior of its inverse system can ideally be characterized by Volterra series expansion (28). However, the full-scale Volterra series expansion requires estimate of multi-variate kernel functions which may be computationally impractical to implement and its estimation might be slow in convergence. In this research, we adopted a simplified version called one-dimensional Volterra filters (ODVF) (22) — assume that the inverse system can be modeled as follows,


(7) $$\begin{array}{l} y\left[n\right]=\overset{M_{1}-1}{\underset{i=0}{\sum }}h_{1}\left[i\right]x\left[n-i\right]+\overset{M_{2}-1}{\underset{i=0}{\sum }}h_{2}\left[i\right]{\left(x\left[n-i\right]\right)}^{2}+\cdots \\ \;+\overset{M_{p}-1}{\underset{i=0}{\sum }}h_{p}\left[i\right]{\left(x\left[n-i\right]\right)}^{p}+\cdots \end{array}$$

where *x*[*n*] and *y*[*n*] denote the input and output of the inverse system, respectively, *h*_*p*_[*i*] denotes the *p*th-order kernel of ODVF, and *M*_*p*_ is the length of the *p*th-order kernel. Since this research focuses on canceling a cubic distortion, a partial ODVF retaining only the 1st-order and 3rd-order kernels was used; that is,


(8) $$\begin{array}{l} y\left[n\right]\approx \overset{M_{1}-1}{\underset{i=0}{\sum }}h_{1}\left[i\right]x\left[n-i\right]+\overset{M_{3}-1}{\underset{i=0}{\sum }}h_{3}\left[i\right]{\left(x\left[n-i\right]\right)}^{3}. \end{array}$$

The filter coefficients could be obtained by the adaptive least mean square (LMS) method (29). The LMS method involves updating the filter coefficients constantly as the time index *n* proceeds. First, denote the filter coefficients in the following vector form,


$${\text{h}}_{1,n}={\left(h_{1}\left[0\right],h_{1}\left[1\right],\ldots ,h_{1}\left[M_{1}-1\right]\right)}^{T}\in {\mathbb{R}}^{M_{1}},$$

and similarly,


$${\text{h}}_{3,n}={\left(h_{3}\left[0\right],h_{3}\left[1\right],\ldots ,h_{3}\left[M_{3}-1\right]\right)}^{T}\in {\mathbb{R}}^{M_{3}}.$$

Note that the subscript *n* indicates that both vectors are updated as *n* increases. Then, an approximation error signal can be defined as follows,


(9) $$\begin{array}{l} e\left[n\right]=d\left[n\right]-y\left[n\right]\approx d\left[n\right]-{\text{h}}_{1,n}^{T}\text{x}\left[n\right]-{\text{h}}_{3,n}^{T}{\text{x}}_{3}\left[n\right], \end{array}$$

where *d*[*n*] is a desired signal to be defined shortly, and vectors **x**[*n*] and **x**_3_[*n*] are defined as follows,


$$\text{x}\left[n\right]={\left(x\left[n\right],x\left[n-1\right],\ldots ,x\left[n-M_{1}+1\right]\right)}^{T}\in {\mathbb{R}}^{M_{1}}$$

and


$${\text{x}}_{3}\left[n\right]={\left({\left(x\left[n\right]\right)}^{3},{\left(x\left[n-1\right]\right)}^{3},\ldots ,{\left(x\left[n-M_{3}+1\right]\right)}^{3}\right)}^{T}\in {\mathbb{R}}^{M_{3}}.$$

Finally, the update equations are given as follows,


(10) $$\begin{array}{l} {\text{h}}_{1,n+1}={\text{h}}_{1,n}+\alpha e\left[n\right]\text{x}\left[n\right], \end{array}$$

and similarly,


(11) $$\begin{array}{l} {\text{h}}_{3,n+1}={\text{h}}_{3,n}+\alpha e\left[n\right]{\text{x}}_{3}\left[n\right], \end{array}$$

where α = 6 × 10^−3^ denotes a stepsize that was chosen empirically.

**Figure 6:**
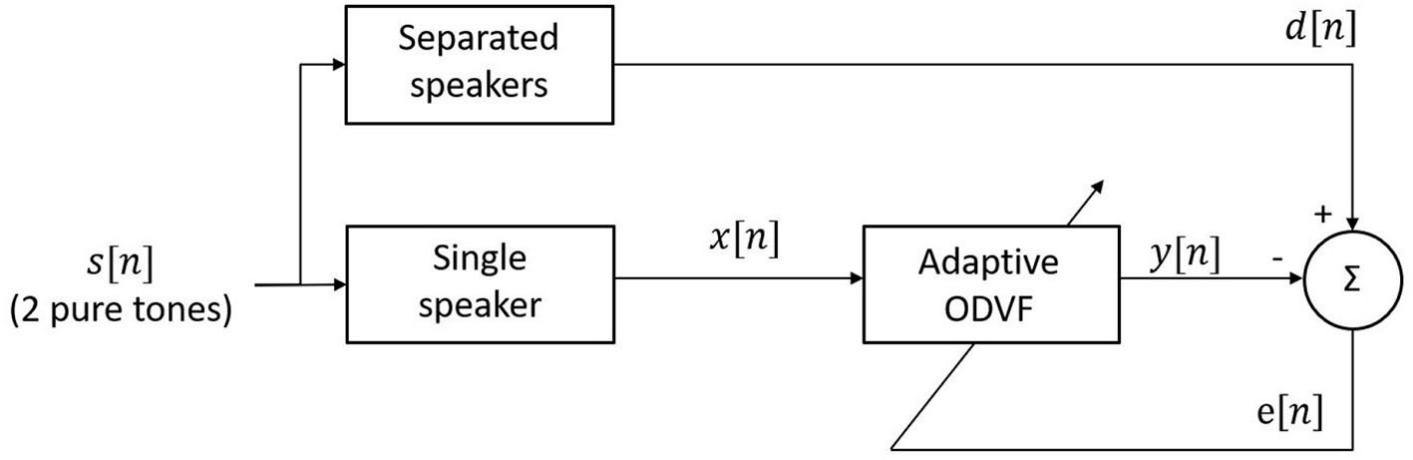
Block diagram for computing the ODVF coefficients.

Figure 6 shows how the ODVF coefficients were obtained in this research. The stimulus *s*[*n*] contained two primary tones, and we set *d*[*n*], the desired signal, to be the signal recorded by sending the two tones to separate speakers of a reliable reference probe, so *d*[*n*] was free of IMD. Meanwhile, the input *x*[*n*] to the ODVF was the signal recorded by using one single speaker while *y*[*n*] denotes the output of the ODVF. Here, we emphasize that the adaptive procedure was performed offline just for one time, and it was not necessary to repeat the procedure when measuring DPOAE from individual ears. In practice, we first recorded *x*[*n*] and *d*[*n*] separately in the same 2-cc syringe with the same stimulus *s*[*n*]. Then, the filter coefficients **h**_1,0_ were initialized at 1 and **h**_3,0_ were initialized at 0, and we computed the updates iteratively according to Equations (9–11). After the filter coefficients converged, we could expect that the variance of *e*[*n*] would be minimized and *y*[*n*] should approximate *d*[*n*], which is a cubic distortion-free signal, in a stochastic sense.

In practice, we found that the 1st-order kernel *h*_1_[*n*] tended to converge to a band-pass filter around the primary-tone frequencies which simply rejected all the intermodulation components linearly. This caused the “training” of **h**_3,*n*_ to fail. Therefore, we set the 1st-order kernel length *M*_1_ to 1 to ensure that *h*_3_[*n*] learns to cancel the cubic distortion.

### 2.4. Equipment

All recordings were collected using a Python script that controls the ER-10C DPOAE probe-microphone (Etymotic Research) system via a 24-bit soundcard (Fireface UFX II, RME). The sampling frequency was set to 48 kHz. All recordings were done in a sound-proof room with the noise floor of approximately 19-21 dB SPL (30).

### 2.5. Human Subjects

Twelve subjects between age 22 and 32 participated in the research, including 8 males and 4 females. The compensation strategy was tested on the data from 5 of the subjects, while the cancellation strategy was tested on the data from 7 subjects. All the subjects did not have ear infection or report any hearing problems at the time of experiment. The recruitment of human subjects was approved by the IRB of National Tsing Hua University (No. 10912HE101).

## 3. Results

Here we report the efficacy of applying the compensation and cancellation strategies.

### 3.1. The Compensation Strategy

We first tested the signal acquisition protocol in a 2-cc syringe. The parameters being tested were β = 1.5, 2.0 or 3.0, and *P*_1_ = *P*_2_ = 6.3, 11.3, and 20.0 mPa, which correspond to 50, 55, and 60 dB SPL, respectively. The primary frequencies were *f*_1_ = 1, 000 and *f*_2_ = 1, 200 Hz. We quantify the performance of IMD3 suppression by the following index,


(12) $$\begin{array}{l} K=20\log_{10}\left|\frac{G_{\text{IMD}3}\left(P_{1},P_{2}\right)}{\beta G_{\text{IMD}3}\left(P_{1},P_{2}\right)-G_{\text{IMD}3}\left(\beta P_{1},\beta P_{2}\right)}\right|. \end{array}$$

Here, *K* is just the IMD3 to IMD3 residual ratio in dB scale.

The values *K* for different combinations of β and *L*_1_ are listed in Table 1. Note that *K* is quite insensitive to change in *L*_1_, while β = 1.5 gives the highest *K*. Therefore, β = 1.5 was chosen for testing in the ear. Also, *L*_1_ = *L*_2_ = 60 dB SPL was selected in order to maximize DPOAE and its residual.

**Table 1:** The suppression index *K* for different combination of parameters.

	***L*_1_ = *L*_2_ (dB SPL)**		
	**50**	**55**	**60**
β = 1.5	21.7	21.9	22.2
β = 2.0	16.2	15.9	16.5
β = 3.0	12.0	13.1	12.4

DPOAEs residuals were recorded from five subjects for their left and right ears using the compensation strategy. Two protocols were considered. The first one is called the “stereo” protocol which uses separate speakers to obtain the ground truth of DPOAE and the residual thereof after applying the compensation strategy. The second is called “mono” protocol and it uses a single speaker mentioned in section 2.2.2 to obtain the DPOAE residual subject to the IMD3 interference. The residual obtained with the stereo protocol should be regarded as a performance upper-bound for the compensation strategy since the IMD3 component is negligible when both speakers are used. We then study the correlation between the residual level obtained by both protocols and the true DPOAE level to evaluate the effectiveness of the compensation strategy.

**Figure 7:**
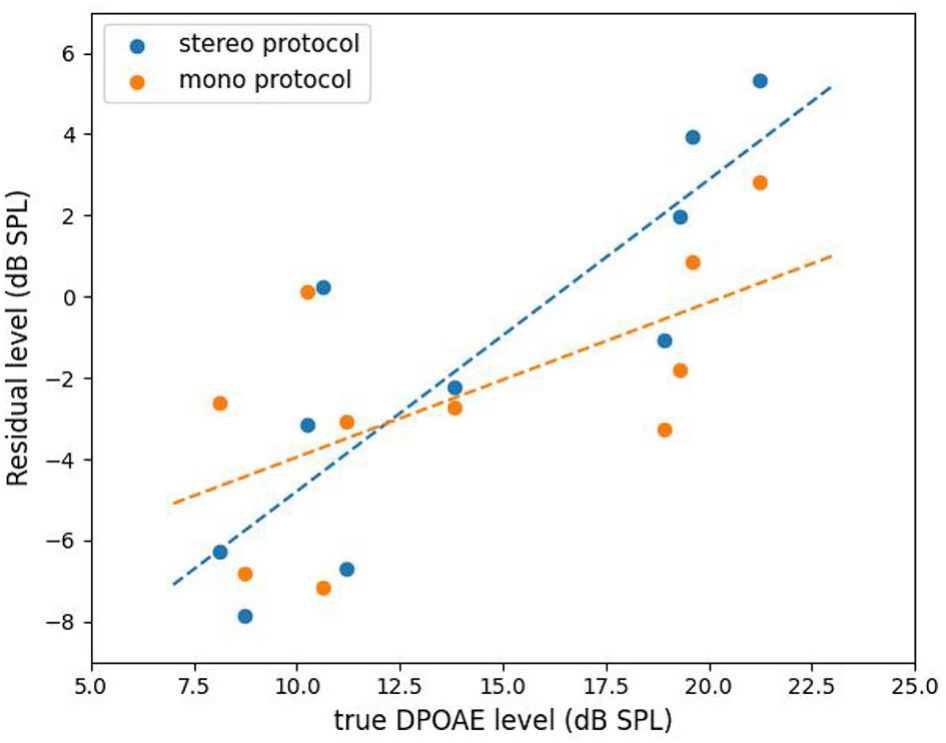
The residual level vs. the true DPOAE level with stereo and mono protocols, respectively. The dashed lines are least-square fits to the data.

The results of mono vs. stereo protocols are plotted in Figure 7. The x-axis is the true DPOAE level obtained with two speakers, and the y-axis represents the DPOAE residual levels obtained with the two protocols. The dashed lines show the results of linear regression. For the stereo protocol, the regression line is *y* = 0.77*x* − 12.46, and for the mono protocol, the regression line is *y* = 0.38*x* − 7.76. By using the stereo protocol, the correlation between DPOAE residual and true DPOAE level, both in dB SPL, equals to 0.86 with a high significance (*p* < 0.001). However, the DPOAE residual levels were 15 dB lower than the real DPOAE levels in average. This indicates that the DPOAE was suppressed by about 80% after applying the compensation strategy. Note that, nevertheless, the IMD3 component was suppressed by 22 dB under the same settings (see Table 1). Thus, we can say that the compensation strategy improved the DPOAE to IMD3 ratio by 7 dB in average. By using the mono protocol, the correlation between the residual and the true DPOAE level is lowered to 0.62. Nevertheless, with a *p* < 0.01, the correlation is deemed significant for this particular set of data, in the sense that the null hypothesis (no correlation) is rejected.

### 3.2. The Cancellation Strategy

**Figure 8:**
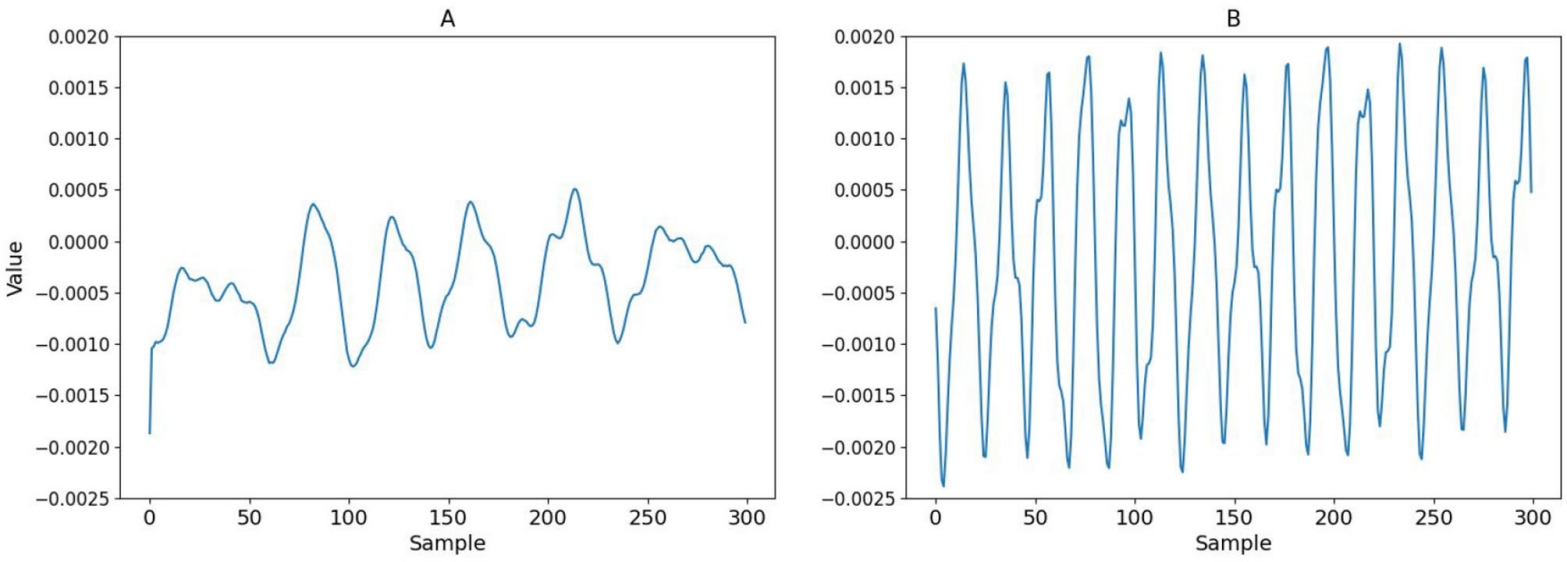
The 3rd-order filter coefficients *h*_3_[*n*] under **(A)** *f*_1_ = 1, 000 Hz and **(B)** *f*_1_ = 2, 000 Hz. The length of the filter was set to 300, which corresponds to 6.25 ms at 48 kHz sampling rate.

We obtained different sets of ODVF coefficients at *f*_1_= 1,000, 1,200, 1,500, and 2,000 Hz, respectively, while *f*_2_/*f*_1_ = 1.2 was fixed. The amplitude of both tones was set to 20 mPa (60 dBSPL), and the recordings ran for 10.5 s with 2.5 ms raised cosine ramp for the rising and falling edges of the stimulation. Empirically, the recording time was sufficiently long to ensure convergence of the filter coefficients. Figure 8 shows the resulting 3rd-order filter coefficients *h*_3_[*n*] for *f*_1_= 1,000 and 2,000 Hz, respectively. These *h*_3_[*n*] coefficients were subsequently used for validating the proposed cancellation strategy depicted in Figure 5.

**Figure 9:**
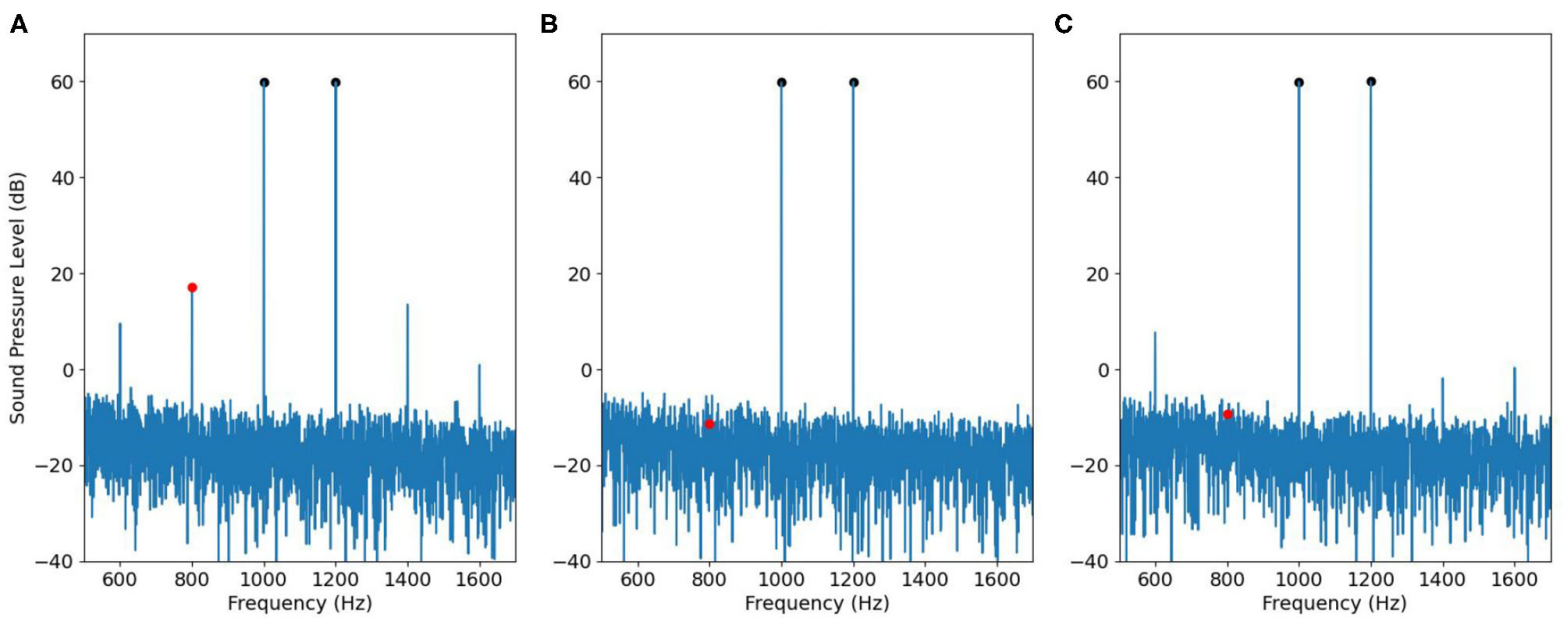
Effectiveness of ODVF in canceling IMD3 in a syringe, visualized in the frequency domain. **(A)** The signal recorded by using a single speaker. **(B)** The signal recorded by using two speakers. **(C)** The signal recorded by using the adjusted input signal in Figure 5.

Following the workflow described in Figure 5, we first tested the adjusted input signal in the 2-cc syringe. The results are shown in Figure 9. Figure 9A is the signal recorded by using a single speaker to play two primary tones. Therefore, the spectrum contains intermodulation distortion. Figure 9B is the signal recorded by using separated speakers and it therefore does not contain intermodulation distortion, and Figure 9C is the signal by using the adjusted input signal produced by the workflow of Figure 5. The black dots represent the magnitude of primary tones *f*_1_ and *f*_2_, the red dot represents the magnitude of the IMD3. All the three recordings ran for 2.5 s with 2.5 ms raised cosine ramp for the rising as well as falling edges of the stimulation. The result shows that IMD3 is largely reduced to submerge below the noise floor. Note that the 2*f*_2_ − *f*_1_ component at 1400 Hz is also suppressed, though not as perfectly as at 2*f*_1_ − *f*_2_. The fifth-order distortions at 600 and 1600 Hz remain unchanged.

**Figure 10:**
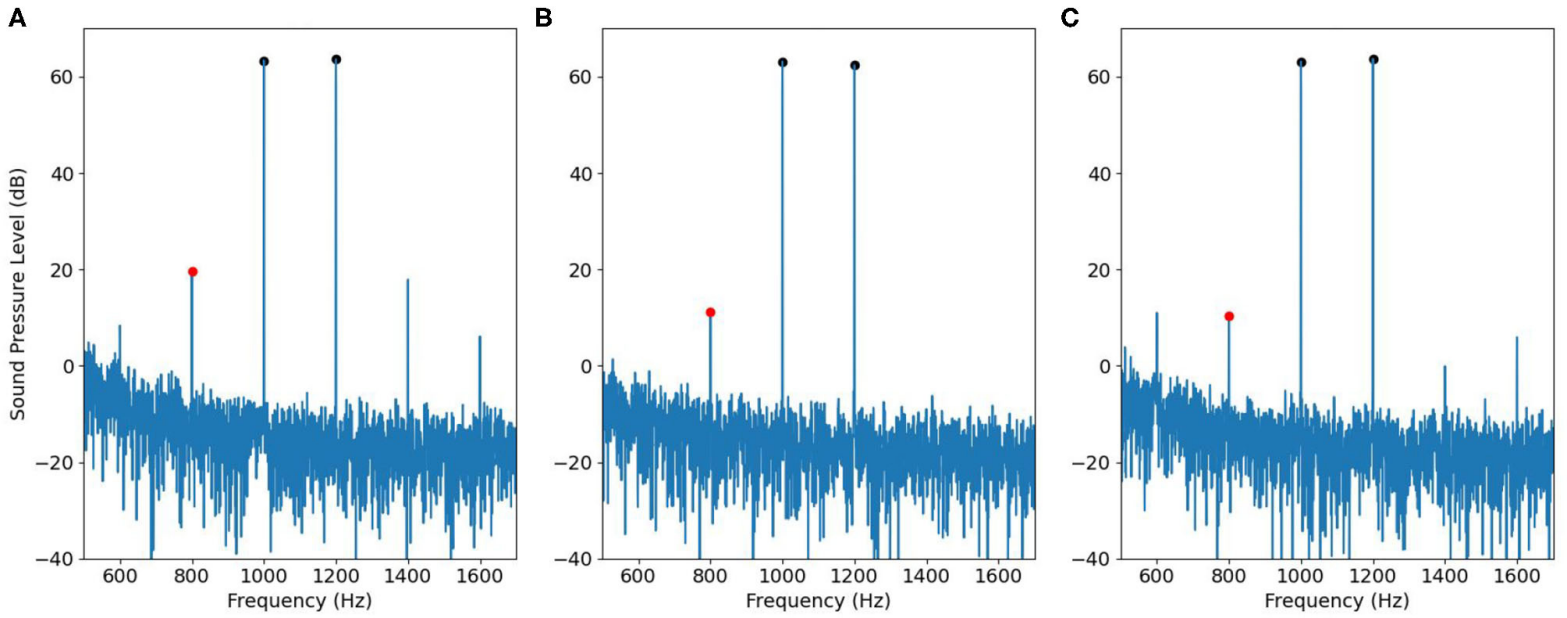
An example of DPOAE estimation by the cancellation strategy when *f*_1_ is 1,000 Hz, viewed in the frequency domain. **(A)** The signal recorded by using a single speaker. **(B)** The desired signal recorded by using two speakers. **(C)** The signal recorded by using adjusted input signal in Figure 5.

**Figure 11:**
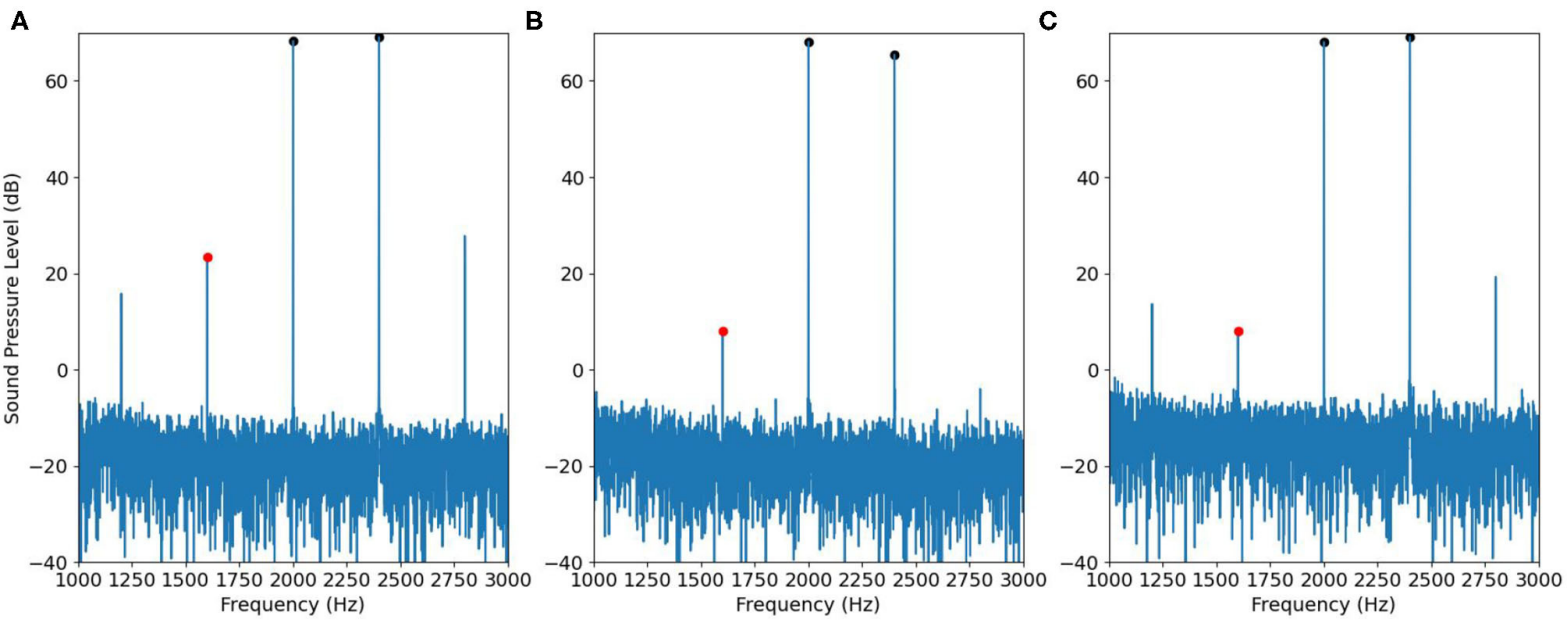
Similar to Figure 10, but with *f*_1_= 2,000 Hz.

Then we applied the same recording procedure in human ears. Figures 10, 11 show some typical results. Figure 10A contains DPOAE interfered with the original IMD3, Figure 10B shows the ideal signal recorded by using separate speakers for the primary tones, and the results are regarded as the true DPOAE signal to compare against. Figure 10C is the signal recorded by using the adjusted input signal described in Figure 5; the signal contains DPOAE interfered with the remaining IMD3.

**Figure 12:**
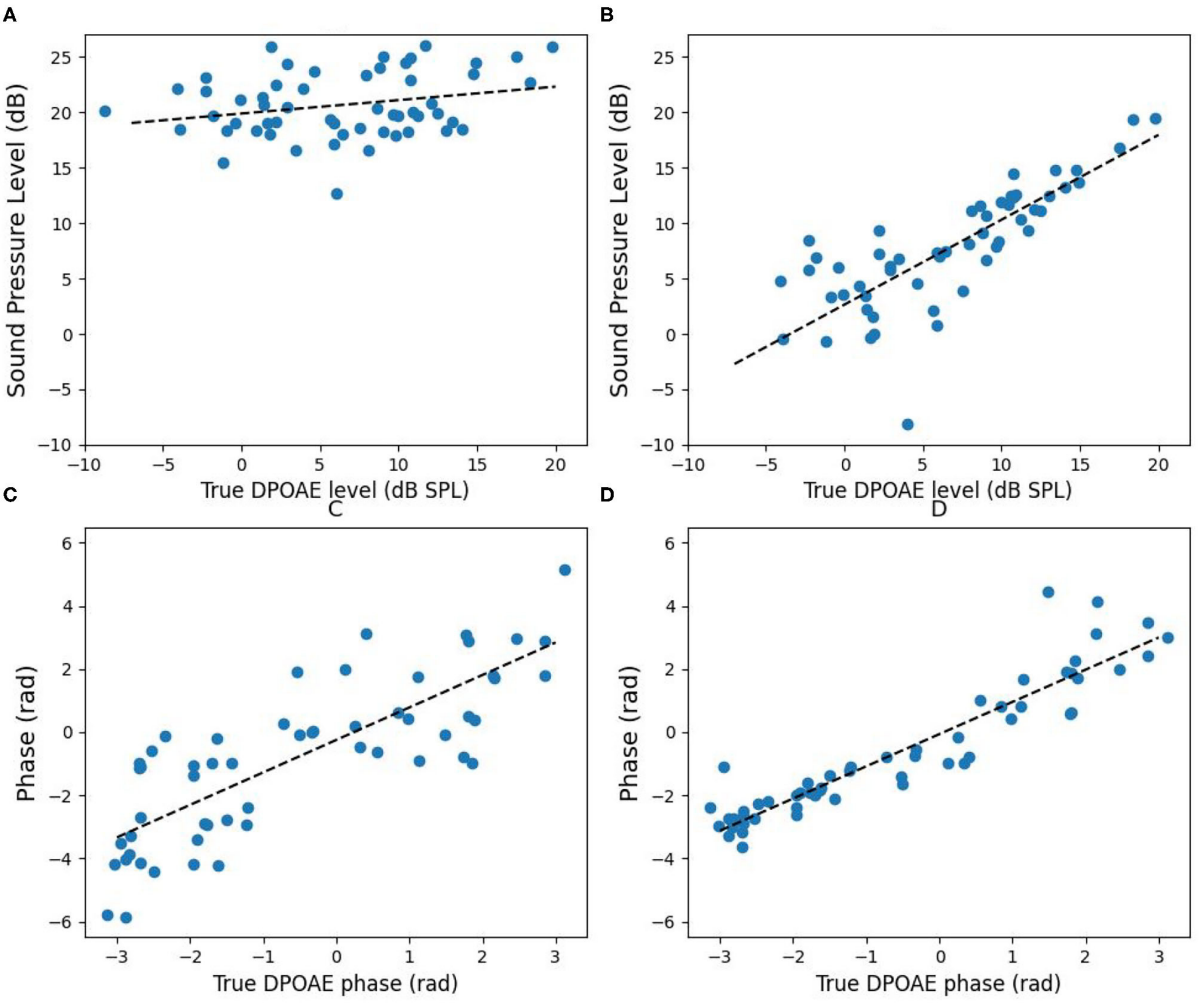
Comparison of DPOAE data without and with the IMD3 cancellation strategy. **(A)** The sound pressure level without applying the IMD3 cancellation strategy; **(B)** the sound pressure level after applying the IMD3 cancellation strategy; **(C)** the phase without, and **(D)** the phase with the IMD3 cancellation strategy. In each plot, the dashed line shows the result of linear regression.

We also extended the experiments to *f*_1_ = 1, 200, 1, 500, and 2, 000 Hz while *f*_2_/*f*_1_ = 1.2. The results without (Figures 12A,C) and with (Figures 12B,D) the IMD3 cancellation strategy are shown in Figure 12. In Figures 12A,B, the horizontal axis is the true DPOAE level, and the vertical axis is the magnitude at *f*_DP_. In Figures 12C,D, the horizontal axis is the true DPOAE phase, and the vertical axis is the phase measured at *f*_DP_. The linear regression lines are also shown for visualization.

Without applying IMD3 cancellation strategy, the regression lines of the magnitude and the phase are *y* = 0.12*x* + 19.87 and *y* = 1.03*x* − 0.25, respectively, and the root mean square errors (RMSE) are 15.52 dB and 1.50 rad, respectively. After applying IMD3 cancellation strategy, the regression lines of the magnitude and the phase become *y* = 0.76*x* + 2.64 and *y* = 1.02*x* − 0.06, respectively, and the RMSE are reduced to 3.88 dB and 0.76 rad, respectively.

After applying the IMD3 cancellation strategy, we calculated the correlation between the estimated and the true DPOAE sound pressure levels, and the correlation between the estimated and the true DPOAE phase, respectively. Subsequently, we ran the Wald test with t-distribution to evaluate the significance of correlation. The correlation between the estimated and true DPOAE levels equals to 0.81 with a high significance (*p* < 0.001); also, the correlation between the estimated and the true DPOAE phase equals to 0.93 with a high significance (*p* < 0.001).

In contrast, without applying the IMD3 cancellation strategy, the correlation between the measured DPOAE and the true DPOAE sound pressure levels equals to 0.25 with a low significance (*p* = 0.057). Although the phase estimation error is higher without applying the cancellation strategy, the cross-correlation between the measured and the true DPOAE phase was still high (0.80) and significant (*p* < 0.001).

The number of data points turns out to be 55. We recruited 7 subjects, test both ears at 4 frequencies, resulting in 56 DPOAE magnitudes and phases. However, the DPOAE level of one of the ears was below the noise floor when *f*_1_ = 1, 000 Hz. So that single point of data was abandoned.

## 4. Discussion

As true wireless, noise-cancellation earphones are gaining popularity in recent years, the ear canal also becomes an over-booked space for various body sensors to enter and make the earphone intelligent (31, 32). Since active noise-cancellation earphones are indeed equipped an internal microphone^1^, there is no reason why the microphone cannot measure DPOAE. The main factor that hinders such application might be the interference due to loudspeaker IMD. As much as we are aware of, consumer earphones are not typically designed to have two speakers in one ear^2^, so sending the primary tones to separate speakers would not be a choice. In this research, we demonstrated that “wrongly” using one single loudspeaker to play the *f*_1_ and *f*_2_ tones may still work as long as we can cancel the IMD3 it generates. Hence, we hope that this research can serve as a feasibility study for the earphone industry to promote DPOAE as a service to consumers of active noise cancellation earphones. We envision that making DPOAE available at home could also enrich any remote hearing care program in the future.

Among the two proposed strategies, cancellation outperforms compensation in producing a more accurate prediction of the true DPOAE level. On one hand, the cancellation strategy achieves a higher cross-correlation to the true DPOAE level (0.81) than the compensation strategy (0.62); one the other hand, it also provides a direct estimate of the DPOAE magnitude and phase, instead of just a residual. It remains to be seen in the future whether similar results would be obtained with a larger sample size.

The usage of Volterra filters also brings up many research questions. For instance, Figure 8 shows that the 3rd-order function *h*_3_[*n*] are different for different choices of (*f*_1_, *f*_2_). Presently, we are uncertain whether (a) this is a limitation due to omitting all the off-diagonal elements of the Volterra filter to make it one-dimensional, or (b) is it possible to apply certain transformation akin to pre-whitening so the adaptive system eventually “learns” a universal ODVF for canceling all the cubic distortions given any input signal. Apparently, there is still much room to explore on this topic.

Meanwhile, as much as the compensation strategy is concerned, it is surprising that we found a large region on the (*L*_1_, *L*_2_) plane where the IMD3 level grows *quasi-linearly* when *L*_1_ and *L*_2_ increase proportionately. In practice, it may be interesting to see if a similar property can be observed in other DPOAE probes or consumer earphones. We speculate that the quasi-linear growth is an epiphenomenon because cubic distortion is by nature a third-order component. Under the light of Taylor expansion in Equation (2), we can expect IMD3 to demonstrate a 3 dB/dB growth when the stimulus is at low intensity—and so does DPOAE (23). As the intensity of η increases, higher order terms *G*^(*k*)^(0)η^*k*^/*k*! begin affecting the growth function. In particular, all the odd-order terms should jointly reduce the slope of growth of *B*_2,−1_ and account for its saturation (it is straightforward to show that the even-order terms do not contribute to *B*_2,−1_). So the fact that we observe nearly 1 dB/dB growth at the working range of (*L*_1_, *L*_2_) may just be a coincidence.

Some other techniques might be worth consideration for estimating the DPOAE level under IMD3 interference. As mentioned in section 1, DPOAE itself has two components — the direct one and the reflection. Based on the difference in latency, Vetesník et al. (21) designed short pips to elicit DPOAE and applied envelope tracking techniques to *separate* the two components. If the loudspeaker distortion is generated within a shorter time before DPOAE emerges, one might be able to identify an early peak by tracking the instantaneous amplitude at the 2*f*_1_ − *f*_2_ frequency. Thus, the loudspeaker IMD3 and DPOAE might be separated in the time domain. This may require careful re-thinking of the stimulus design and is left for future exploration.

## 5. Conclusions

We proposed two strategies to estimate the DPOAE level subject to interference from the loudspeaker IMD3. The compensation strategy was designed to suppress IMD3 based on its quasi-linear growth with respect to primary-tone levels, in contrast to DPOAE's sub-linear growth. Results show that, although the DPOAE level was also suppressed by 80%, the residual level correlates to the true DPOAE level. The cancellation strategy adjusted the input signal nonlinearly to emulate distortion-free stimulation. It thus recovered both the DPOAE magnitude and phase directly. Overall, this research suggests that it might be feasible to use a single-loudspeaker probe to measure DPOAE. Testing with a larger sample of human subjects as well as various types of earphones shall ensue to evaluate whether commercial noise-cancellation earphones could be utilized to allow sufficiently accurate DPOAE measurement at home.

## Data Availability Statement

The raw data supporting the conclusions of this article will be made available by the authors, without undue reservation.

## Ethics Statement

The studies involving human participants were reviewed and approved by Research Ethic Committee, National Tsing Hua University. The patients/participants provided their written informed consent to participate in this study.

## Author Contributions

YCC discovered the almost linear growth in IMD3, and came up with the compensation strategy. WCH tested the compensation strategy, and conceived the cancellation strategy using ODVF and then implemented and tested the cancellation strategy. YWL supervised the research and is responsible for the manuscript preparation process. All authors contributed to the article and approved the submitted version.

## Conflict of Interest

The authors declare that the research was conducted in the absence of any commercial or financial relationships that could be construed as a potential conflict of interest.

## Publisher's Note

All claims expressed in this article are solely those of the authors and do not necessarily represent those of their affiliated organizations, or those of the publisher, the editors and the reviewers. Any product that may be evaluated in this article, or claim that may be made by its manufacturer, is not guaranteed or endorsed by the publisher.

## Footnotes

^1^For example *AirPods Pro and AirPods Max Active Noise Cancellation and Transparency mode*, https://support.apple.com/en-us/HT210643.

^2^For example “Qualcomm QCC3056 is an ultra-low power, single-chip solution, optimized for use in truly wireless earbuds and hearables.” The chip features mono audio playback. https://www.qualcomm.com/products/qcc3056.
